# Supplementary material for: Automated cell counting for Trypan blue-stained cell cultures using machine learning
Source: PLoS One. 2023 Nov 28;18(11):e0291625. doi: 10.1371/journal.pone.0291625 (PMC10684072; doi:10.1371/journal.pone.0291625)
Supplement: S1 File — (DOCX) [file pone.0291625.s001.docx]

# Supplementary Information for:

Automated cell counting for Trypan blue-stained cell cultures using machine learning

**Louis Kuijpers^1,2^, Edo van Veen^1^, Leo A. van der Pol^2^, and Nynke H. Dekker^1,*^**

^1^ Delft University of Technology, Van der Maasweg 9, 2629 HZ Delft, The Netherlands

^2^ Intravacc B.V., Antonie van Leeuwenhoeklaan 9, 3721 MA Bilthoven, The Netherlands

* Corresponding author:

E-mail: n.h.dekker@tudelft.nl

Several online resources accompany this publication as described in this Supplementary Information file:

- The full ML model as well as a guide on how to install and use it (Van Veen E., ML model code and documentation; available at: <https://gitlab.tudelft.nl/nynke-dekker-lab/public/cell-counter> or as persistent URL: <https://gitlab.tudelft.nl/nynke-dekker-lab/public/cell-counter/-/tree/3cb1e0165012402914c5bbdb42179fae0eadcc24>).
- The annotated test and training data sets, supplied in a 4TU repository (Kuijpers L, Van Veen E, Van der Pol L, Dekker NH. Code and train, validation, test data sets underlying publication: Automated cell counting for trypan blue-stained cell cultures using machine learning. 2023. Available at: https://doi.org/10.4121/21695819).
- The same 4TU repository also includes the confusion matrices used to derive the performance parameters as well as the complete set of evaluated images (n = 122 for insect cells and n = 52 for HEK cells).
