# Supplementary material for: Automated cell counting for Trypan blue-stained cell cultures using machine learning
Source: PLoS One. 2023 Nov 28;18(11):e0291625. doi: 10.1371/journal.pone.0291625 (PMC10684072; doi:10.1371/journal.pone.0291625)
Supplement: Attachment — Submitted filename: Kuijpers et al point-by-point response.pdf [file pone.0291625.s002.pdf]

Delft, August 24th, 2023

We thank the reviewers for their willingness to review our manuscript and appreciate their positive evaluation and suggestions. We have addressed the raised questions and concerns by extending the explanations and discussion in our revised protocol. Please find below (in blue) our point-by-point response to the reviewers' questions and comments, as well as a brief listing of the changes made in the revised manuscript version (in red). Textual changes in the revised manuscript are marked using track changes.

Kind regards,
The authors

Journal requirements

When submitting your revision, we need you to address these additional requirements.

1. Please ensure that your manuscript meets PLOS ONE's style requirements, including those for file naming. The PLOS ONE style templates can be found at https://journals.plos.org/plosone/s/file?id=wjVg/PLOSOne_formatting_sample_main_body.pdf and https://journals.plos.org/plosone/s/file?id=ba62/PLOSOne_formatting_sample_title_authors_affiliations.pdf

Response: We have updated the document with the following alterations to meet the PLOS One format:

- The title and authors affiliations
- Major section headings to level 1, 18pt font, and bold
- Sub-section headings to level 2 headings, 16pt font and bold
- Change all figure captions and cross-references from "Figure" to "Fig"
- Manuscript changed to double-space paragraph format
- Deleted funding interest from acknowledgments
- Update citation style
- Moved the supplemental information from above to below the references

2. Please note that PLOS ONE has specific guidelines on code sharing for submissions in which author-generated code underpins the findings in the manuscript. In these cases, all author-generated code must be made available without restrictions upon publication of the work. Please review our guidelines at https://journals.plos.org/plosone/s/materials-and-software-sharing#loc-sharing-code and ensure that your code is shared in a way that follows best practice and facilitates reproducibility and reuse.

Response: Code will be made available in the previously cited link to the website, which has its own documentation that details information needed to run the code, data availability statement, and clear license indication (MIT license). Additionally, we have added the persistent URL as requested by PLOS One for code sharing. If accepted we will make all data open using the indicated repositories. However, for actually publishing the data using the repositories stated in the manuscript we require the DOI of the metadata (the published manuscript).

3. Thank you for stating the following in the Acknowledgments Section of your manuscript:

"We thank Bèr van der Wijden for providing the HEK cells for imaging, Abbas Freydoonian for maintaining the insect cell cultures, and Oussama Akhiyat for aiding in the code development. We gratefully acknowledge funding from Intravacc B.V. to L.v.d.P. and N.H.D."

We note that you have provided funding information that is currently declared in your Funding Statement. However, funding information should not appear in the Acknowledgments section or other areas of your manuscript. We will only publish funding information present in the Funding Statement section of the online submission form.

Please remove any funding-related text from the manuscript and let us know how you would like to update your Funding Statement. Currently, your Funding Statement reads as follows:

“Recipients: L.v.d.P. and N.H.D.
Grant name: COMET
Grant number: none
Funder: Intravacc B.V.
Funder website: https://www.intravacc.nl/
The funders had no role in study design, data collection and analysis, decision to publish, or preparation of the manuscript.”
Please include your amended statements within your cover letter; we will change the online submission form on your behalf.
Response: We have deleted the funding-related text from the manuscript, agree with the updated statement, and have added the funding statement to the cover letter.

4. We note that you have stated that you will provide repository information for your data at acceptance. Should your manuscript be accepted for publication, we will hold it until you provide the relevant accession numbers or DOIs necessary to access your data. If you wish to make changes to your Data Availability statement, please describe these changes in your cover letter and we will update your Data Availability statement to reflect the information you provide.
Response: If accepted, we will make all data open using the indicated repositories. However, for actually publishing the data using the repositories stated in the manuscript, we require the DOI of the metadata (contained in the published manuscript). The presented DOI is a reserved DOI, which will be made available as soon as you provide us with the metadata’s DOI.

5. We note that Figure 1 in your submission contain copyrighted images. All PLOS content is published under the Creative Commons Attribution License (CC BY 4.0), which means that the manuscript, images, and Supporting Information files will be freely available online, and any third party is permitted to access, download, copy, distribute, and use these materials in any way, even commercially, with proper attribution. For more information, see our copyright guidelines: http://journals.plos.org/plosone/s/licenses-and-copyright.
We require you to either (1) present written permission from the copyright holder to publish these figures specifically under the CC BY 4.0 license, or (2) remove the figures from your submission:
a. You may seek permission from the original copyright holder of Figure 1 to publish the content specifically under the CC BY 4.0 license.
We recommend that you contact the original copyright holder with the Content Permission Form (http://journals.plos.org/plosone/s/file?id=7c09/content-permission-form.pdf) and the following text:
“I request permission for the open-access journal PLOS ONE to publish XXX under the Creative Commons Attribution License (CCAL) CC BY 4.0 (http://creativecommons.org/licenses/by/4.0/). Please be aware that this license allows unrestricted use and distribution, even commercially, by third parties. Please reply and provide explicit written permission to publish XXX under a CC BY license and complete the attached form.”
Please upload the completed Content Permission Form or other proof of granted permissions as an "Other" file with your submission.
In the figure caption of the copyrighted figure, please include the following text: “Reprinted from [ref] under a CC BY license, with permission from [name of publisher], original copyright [original copyright year].”
b. If you are unable to obtain permission from the original copyright holder to publish these figures under the CC BY 4.0 license or if the copyright holder’s requirements are incompatible with the CC BY 4.0 license, please either i) remove the figure or ii) supply a replacement figure that complies with the CC BY 4.0 license. Please check copyright information on all replacement figures and update the figure caption with source information. If applicable, please specify in the figure caption text when a figure is similar but not identical to the original image and is therefore for illustrative purposes only.
Response: We have obtained the publishing license as requested, and it has been added to the submission under “Other”.

Reviewer comments:

Reviewer #1

We thank the reviewer for the positive evaluation of our protocol manuscript. We have revised the manuscript to accommodate the points raised.

The authors present ML-based application for automated cell counting that provides high accuracy at low cost. This research is significant since manual count using traditional approaches such as hemocytometer is often unreliable and not reproducible as results may vary when analyzed by different operators. The manual binary classification live or dead is often hard to make since sometimes cells are not just "white" and "blue" but in between and some operators may call it live and some dead.
I appreciate the efforts done by the authors of this manuscript since they are aimed to solve a problem for counting insect cells that lack quantitative and reliable tools. They also aim to avoid buying expensive technology, which is highly valuable. Overall, the work presented is technically sound and promising. I only have concerns that they seemed to verify their model using manual human visual approach which they criticized themselves. I think their manuscript deserves publication, but needs to address at least the following:
1) how many operators verified the model manually and if there was 100% consensus or not,
Response: Please see point 4 under suggestions/major concerns, below.

2) It seems that there are out-of-focus cells in the pictures. Could a different, thinner chamber be used to make sure all the cells are in the same focus plane?
Response: We appreciate the reviewer's perceptive observations made regarding the images showcased in the paper. However, we would like to clarify that the scenario described by the reviewer does not apply to the images shown. All the cells present in the chambers are in the same focal plane. What the reviewer interprets as out-of-focus cells are actually artifacts that result from contamination on the microscope (scratches/dust/etc.). We deliberately retained this data in the images, recognizing it as an opportunity to assess the capacity of the machine learning algorithm to potentially misclassify such instances.

To clarify this point, we have added the following sentence at line 278 of the revised manuscript: "Additionally, anomalies originating from microscope contamination, such as scratches or dust, were not classified as cells."

3) It would be best if they tested or trained control samples, dead cell control sample, for example, is easy to prepare, since they can use heat-shock or fixation protocols to produce stable dead cells. I think this is far more important than mixing cultures, though, I appreciate the value of the mixed cell population as well.
Response: Please see point 3 under suggestions/major concerns, below.

4) It seems that model can be improved by training close to equal # of live and dead cells to avoid issues discussed in the manuscript (model was able to detect alive cells better than dead cells). Why was it not done if they recognized the issue?
Response: Please see point 4 under suggestions/major concerns, below.

5) I was not clear if their approach solved the aggregation problem. It seems that it did not, but they emphasized that it is a problem and it was my understanding that they are also trying to solve that issue with their ML technique.
Response: see point 5 suggestions/major concerns, below.

Suggestions/major concerns:

1) The title of the manuscript is about cell counting and trypan blue assay. Thus, the introduction should start by introducing state-of-the art cell counting and any issues with the measurements that the authors are aiming to solve. I was lost how for a bit reading about protein expression etc since they didn't link it to cell counting until the fourth paragraph.
Response: We thank the reviewer for pointing out the need to emphasize the most critical message in the introduction. In response to the reviewer's valuable feedback, we have significantly reduced the introduction by omitting unnecessary explanations of the expression platforms, which did not contribute substantially to the manuscript. We have condensed and summarized the text while retaining essential references to allow readers to access underlying information related to the platforms. Additionally, we have relocated the section that elaborates on the determination of cell lysis to the discussion.

2) Figure 1, Machine learning approach, it is unclear what is the sample. Did the authors use a hemocytometer and brightfield images for the analysis or the chambers for automated cell counters. Later the information can be found in the methods, but Fig.1 comes earlier than the explanation. I suggest adding at least a cartoon image of the slide+microscope to the ML method part like for the others.
Response: We express our gratitude to the reviewer for providing valuable feedback regarding the need for further clarification of Figure 1 and the overall approach. In response to the reviewer's suggestion, we have updated Figure 1 to clearly illustrate that both the automated cell counters and the machine learning model require a chamber or liquid container with a known volume for accurate cell counting. Moreover, we have carefully considered the reviewer's recommendation to enhance the depiction by including a microscope. Accordingly, we have incorporated a zoom-in into Figure 1 to represent the microscope used in the cell counting process.

Updated Figure 1:

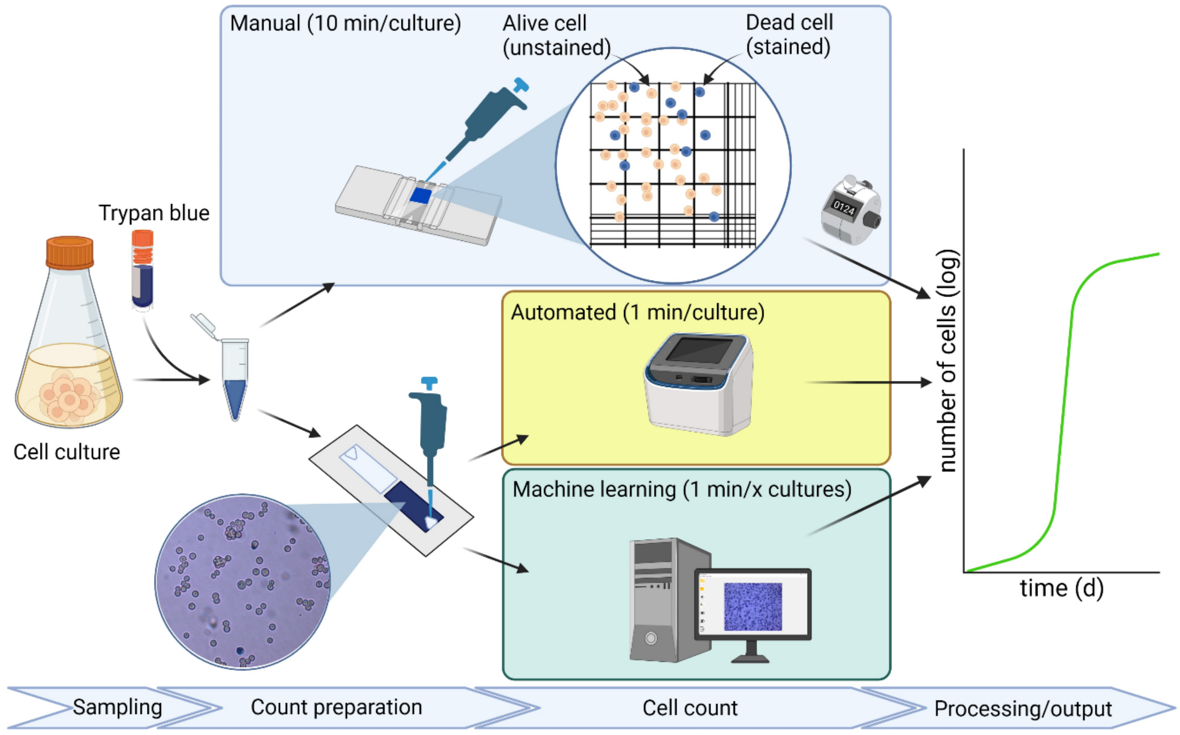

3) For the training of the model, wouldn't it be beneficial to prepare control samples of healthy cultures (with only few dead cells) and 100% dead cell control sample by treating them with a known cell killing methods (e.g.

heat-shock or fixation)? Also, for the training of the model, there is disproportion between the live cell # and dead cell #. The ML experts typically tell me that we need to train equal number of instances in each class to avoid imbalances/bias in the training. This limitation is pointed out later on line 260 “For both cell lines jointly, the model was able to detect alive cells (Figure 4A) slightly better than dead cells (Figure 4B), a consequence of their greater consistency in size and shape and the larger number of alive cells in the training data set.”
Response: We express our gratitude to the reviewer for his/her astute observations that relate to the different aspects used as training data. Although we recognize the prospect of further improving the model’s performance through extended training data with more dead cells:

- It is noteworthy that the misclassification of dead cells is infrequent. This is exemplified by the highly similar F1 scores for dead and alive cells (Figure 4). Hence, the potential bias stemming from an increased number of viable cells in the training dataset does not appear to significantly impact the performance of the ML model.
- We believe that further training may not be necessary, as we wish to underscore that the model exhibits commendable performance, even on cell types it was not originally trained on, such as HEK cells.

4) The model seemed to be validated by visual inspection. However, visual manual approach was criticized earlier in the manuscript. Authors should at least visually verify it with more than one operator. Also, the model could be verified using control 100% dead samples (or other control samples).
Response: We thank the reviewer for pointing out that the visual inspection seems counterintuitive, since it is the problem that we are trying to solve. We agree with the reviewer that a single verification might be insufficient. Therefore, we have incorporated an additional visual manual inspection to exclude substantial operator-to-operator variance. Confusion matrices of all evaluations have been added to the manuscript as supplemental information. The text has been updated accordingly:

Line 216 of the revised manuscript: “… to multiple independent manual inspections of all 122 classified …”

Also, near-dead samples of the insect cells (viabilities < 20% and < 10% for Hi5 and Sf9) prepared using the reviewer’s suggested heat-shock method were already present in the original manuscript.

5) It is not clear to me how did their ML tool fix aggregates issue at high cell densities, which they mention is a problem. How was the model verified since visually it is hard to count how many cells are in an aggregate?
Response: We sincerely appreciate the reviewer for bringing up this matter. However, we note that within the paper, we already explicitly acknowledged our inability to address this issue through the ML tool. When cells are significantly aggregated, it is reasonable to expect that the ML model, much like manual or automated cell counters, may encounter challenges in accurately counting them.

We have provided elaboration on this aspect in the manuscript to enhance clarity and understanding:
-Line 299 of the revised manuscript: “High accuracies were also achieved by the ML model for dead cell counts for Hi5 (Error! Reference source not found.**C**) and Sf9 (Error! Reference source not found.**D**) cells, with a maximum overestimation of ~4.5% for the higher cell counts (>150 cells/FoV), **originating from the double counts of aggregated cells**. **Aggregated cells can result in a significant overestimation of cell counts** due to the lack of cell boundary detection, leading to double counts. This is in line with the decreased accuracy observed for heavily aggregated cultures by manual cell counting methods and the majority of automated cell counters.”
-And line 358 of the revised manuscript: “This allows one to apply the ML model to cell cultures with a high cell density without the need for additional dilution prior to analysis, thus reducing additional experimental error and process time, **provided that aggregation is not apparent**.”

6) I cannot comment on Model Performance section since I am not an expert in ML field.

Response: We thank the reviewer for taking the time and effort to assess the manuscript and evaluate its scientific soundness.

7) Data availability: the authors indicated that references 33 and 40 provide the readers the full ML model, access to evaluated images and training data set. However, Ref. 33 website gives an error “DOI not found” and Ref. 40 is not accessible since it asks for “Delft University of Technology GitLab server” login credentials.
Response: We thank the reviewer for pointing out that both of the repositories are currently unavailable. In response to the reviewer’s concerns, we have removed ref 40 from the manuscript. Additionally, we intend to make the code available under the repository referenced in citation 33 of the original manuscript. We regretfully inform the reviewer that we can only publish the repository once the manuscript is accepted, and we obtain the DOI for the publication (which required for publishing the repository). However, we assure the reviewer that we are fully committed to transparency and reproducibility. Therefore, if the reviewer wishes to evaluate the soundness of the code or the ML model in its entirety, we are more than willing to provide all the necessary information and code for his/her assessment.

Reviewer #2

Finding ways to reduce the cost of scientific research without losing its quality is an important task. This is especially true for countries with weak economies, where scientists often do not have access to expensive equipment, including automatic cell counting systems. Based on the results of their research, the authors of the article proposed a convenient, inexpensive method of automatically counting of cells instead of labour-intensive manual counting. The positive thing is that the proposed method is universal and can be applied to different types of cell cultures. In general, the manuscript submitted for review makes a good impression.
Response: We thank the reviewer for reviewing our protocol and appreciate the positive evaluation.

Reviewer #3

The manuscript under review introduces Machine Learning (ML) model aimed at providing an efficient alternative to manual or automated cell counting in Trypan blue-stained microscopic images of insect and HEK cells. The model claims to offer higher speed, lower cost, and the capacity to process numerous images swiftly. It is intended to be beneficial for cell culture operators across all scales and to simplify protein expression requirements in insect cell production platforms.
Despite the promising potential of this work, I have identified several areas of the manuscript that could benefit from improvement:
Response: We thank the reviewer for reviewing our protocol and appreciate the positive evaluation. We addressed all comments in a point-by-point manner.

1) The manuscript is well-written but the introduction is excessively long and could be better aligned with the main objectives of the study.
Response: We express our gratitude to the reviewer for highlighting the need to prioritize the most important message in the introduction. Please, see point 1 reviewer #1, above.

2) The provided illustration diagram in the article lacks logical reasoning in directly feeding the sample to the machine learning model. To address this issue effectively, it is crucial to integrate a detection mechanism into the process.
Response: We thank the reviewer for highlighting the deficiency in the explanation of the machine learning model. Please, see point 2 reviewer #1, above.

3) Reference [3] seems unrelated to the topic at hand. To improve the article's general coherence, it is advised to reevaluate its relevance or eliminate it.

Response: We thank the reviewer for pointing out that reference [3] was redundant and have eliminated the reference from the manuscript.

4) The ML model that was applied in the study needs to be further explained. It would improve comprehension and provide technical depth to include a detailed discussion of its architecture, important parts, and algorithms.
Response: We express our gratitude to the reviewer for highlighting the absence of comprehensive elucidation concerning the ML model, particularly the underlying YOLOv4 architecture. Our primary objective in crafting the manuscript was to ensure accessibility without delving excessively into the intricacies of the ML domain. Nonetheless, we recognize the possibility of inadvertently alienating readers with an interest in the foundational architecture and algorithms. In agreement with the reviewer's perspective, we concur that the manuscript should indeed encompass a succinct exposition of the model. Accordingly, we have revised the manuscript, in particular in the "Methods" section under the subheading "Baseline ML model". We hope that these revisions address the concerns effectively.

Starting from line 188, the revised manuscript now reads: "The YOLOv4 model consists of three architectural components referred to as the head, neck, and backbone. The backbone is the initial part of the neural network responsible for extracting the feature maps from the input image. In YOLOv4, the backbone is based on the CSPDarknet53 architecture and employs a Cross Stage Partial network (CSPNet) structure to improve information flow and feature extra efficiency in comparison to its predecessor: Darknet53 [22,26]. CSPDarknet53 contains a series of convolutional layers that gradually downsample the input image, capturing features at difference scales and levels of abstraction. The extracted features are then passed to the subsequent stages of the model. The neck is an intermediary component inserted between the backbone and the head of the network. It plays a crucial role in aggregating features from multiple stages of the backbone. In YOLOv4, the neck architecture is designed to enhance feature fusion and information flow. It consists of bottom-up pathways, which capture high-resolution features from different stages of the backbone, and top-down pathways which upsample and fuse these features to create a rich feature representation that is used by the detection head [22,27,28]. The head is the final part of the network responsible for predicting object classes and bounding boxes. It takes the fused and aggregated feature maps from the neck and applies additionally convolutional and fully connected layers to predict the presence of objects, their classes, and the corresponding bounding boxes. The head architecture is designed to accurately locate and classify objects in the image"

5) There are difficulties with availability and retrieval in reference [15].
Response: We express our gratitude to the reviewer for bringing to our attention the difficulty in retrieving reference [15]. We have accordingly revised the citation and provided an alternative reference that conveys the same point while being more readily accessible.

6) The methodology section (lines 143 to 145) lacks clarity on how the cell counts in the training and validation sets were determined.
Response: We thank the reviewer for bringing this to our attention. To address this concern, we have included the term "manually" in the manuscript to clarify that the classification of cells in the ML training and validation data sets requires human intervention. This process of assigning the labels "dead" or "alive" manually, is a prerequisite for the ML model construction process. Despite the manual assessment of cell status, we are confident that the large number of cells used in the training and validation datasets effectively mitigates any bias introduced by the manual classification process. Consequently, the final model's ability to classify cells correctly remains largely unaffected, assuring the reliability of the results.

Additionally, we have incorporated an additional visual manual inspection to exclude substantial operator-to-operator variance. Confusion matrices of all evaluations have been added to the manuscript as supplemental information.

Therefore, Line 214 in revised manuscript now reads: "… were constructed, and the cells were annotated manually as …"

7) In line 171, the use of the term 'large test data' appears misleading as the number of data points is relatively small for machine learning studies.
Response: We extend our appreciation to the reviewer for highlighting that the dataset is, in fact, not as extensive as implied in the text. We sincerely apologize for the misleading statement and have removed the term "large" from the manuscript to accurately reflect the dataset's size.

8) Confusion matrices, which are essential for assessing the performance of ML models, are currently absent from the manuscript and should be included.
Response: We thank the reviewer for pointing out that the confusion matrices underlying the performance metrics are absent from the manuscript. They have been added to the 4TU repository link as supplemental information.

The following sentence was added to the manuscript (section supplemental information):
"Furthermore, the confusion matrices used to derive the performance parameters have also been included in the same repository."

9) Additionally, I suggest that the authors consider adding the following reference, as it could provide relevant and beneficial insights to their study: DOI: 10.1109/JBHI.2022.3203893
Response: We thank the reviewer for his thorough evaluation of not only the current manuscript but also putting it in the light of previous work. Notwithstanding the dissimilarity in the types of cells and the adoption of distinct underlying models and algorithms in the proposed study, we concur with the reviewer's perspective that the referenced work can offer valuable insights to readers. Consequently, we have incorporated the suggested reference into the manuscript, aiming to enhance its informational content.

Therefore, Line 83 in revised manuscript now reads: "… Counting of red blood cells and several other cell lines, however an appropriate model for the counting of insect cells remains lacking [23,28-30]"
